# Different Instruments, Same Content? A Systematic Comparison of Child Maltreatment and Harsh Parenting Instruments

**DOI:** 10.1177/15248380221134290

**Published:** 2022-11-28

**Authors:** Sophia Backhaus, Patty Leijten, Franziska Meinck, Frances Gardner

**Affiliations:** 1 University of Oxford, UK; 2 University of Amsterdam, the Netherlands; 3 University of Edinburgh, United Kingdom; 4 University of Witwatersrand, Johannesburg, South Africa; 5 North-West University, Vanderbijlpark, South Africa

**Keywords:** child abuse, domestic violence–assessment, child abuse–child abusers, child abuse–family issues and mediators

## Abstract

Child maltreatment and harsh parenting both include harmful actions by parents toward children that are physical (e.g., spanking, slapping) or emotional (e.g., threatening, yelling). The distinction between these two constructs, in meaning and measurement, is often unclear, leading to inconsistent research and policy. This study systematically identified, reviewed, and compared parent-reported child maltreatment (*N* = 7) and harsh parenting (*N* = 18) instruments. The overlap in parenting behaviors was 73%. All physical behaviors that were measured in harsh parenting instruments (e.g., spanking, beating up) were also measured in child maltreatment instruments. Unique physical behaviors measured in maltreatment instruments include twisting body parts and choking. All emotional behaviors in maltreatment instruments were included in harsh parenting instruments, and vice versa. Our findings suggest similar, but not identical, operationalizations of child maltreatment and harsh parenting. Our findings can help guide discussions on definitions, operationalizations, and their consequences for research on violence against children.

## Key Points of the Instrument Review

- Seventy-three percent of the parenting behaviors measured in child maltreatment and harsh parenting instruments are the same.
- All physical parenting behaviors included in harsh parenting instruments are also included in child maltreatment instruments.
- Physical behaviors unique to child maltreatment instruments are burning, choking, forcing a child to stand or kneel, tying up, twisting a body part, and withholding a meal.
- All emotional behaviors included in harsh parenting instruments are also included in child maltreatment instruments and vice versa.
- There are no emotional behaviors unique to child maltreatment instruments.
- We found high levels of heterogeneity in the behaviors measured between and within harsh parenting and child maltreatment instruments, indicating that not every instrument focused on each concept respectively includes the same behaviors creating a potential risk in measurement selection.

## Implications for Practice, Policy, and Research

- The findings call for a discussion on whether child maltreatment and harsh parenting should be seen and operationalized as similar or different constructs.
- For practice, it is important to identify which parenting behaviors may or may not distinguish child maltreatment from harsh parenting.
- In policy, it is important to clarify the parenting behavior that was measured in the evidence used to inform policy decisions.
- For research, it is important to agree on how child maltreatment and harsh parenting should be defined and assessed.

Decades of research on violence against children came to five firm conclusions: it has detrimental effects on child development and health, is prevalent, violates children’s rights, can be prevented, and, lastly, is mainly perpetrated by parents (United Nations Children’s Fund [UNICEF], 2014a). Violent parenting is typically measured and described as “harsh parenting” or “child maltreatment.” The overlap versus distinction between child maltreatment and harsh parenting, in meaning and measurement, is often unclear. Instruments play a crucial role in shaping our understanding of constructs, and their causes and consequences. As such, this study systematically analyzed and quantified the overlap (vs. distinction) in content of instruments to measure child maltreatment and harsh parenting.

Child maltreatment and harsh parenting can be measured using observation, administrative data, or self-reports of children, parents, or adults retrospectively. Each measurement type comes with limitations, such as underestimating prevalence when using observation or official reports, ethical concerns when asking very young children, social desirability when asking children or parents, and a risk of recall bias when asking adolescents or adults to reflect back on their childhood (Mathews et al., 2020). Most research on violence against young children uses parent self-report instruments, and there are no instruments available for self-report by young children that are valid and reliable (Devries et al., 2018).

### Defining and Measuring Child Maltreatment

Child maltreatment is commonly defined as

all forms of physical and/or emotional ill-treatment, sexual abuse, neglect or negligent treatment or commercial or other exploitation, resulting in actual or potential harm to the child’s health, survival, development or dignity in the context of a relationship of responsibility, trust or power. (World Health Organization [WHO], 1999, p. 15)

Common maltreating behaviors perpetrated by parents are physical and emotional abuse at home (Devries et al., 2018; UNICEF, 2014b). Understanding of what exactly constitutes child maltreatment varies widely by country legislation (ChildONEurope, 2009; ISPCAN, 2008). Some countries separate corporal punishment from child maltreatment, providing a lawful basis for “reasonable” physical force as a means to discipline a child. These countries prohibit physical beating of a child but not hitting a child on the buttocks with an open hand (e.g., Bulgaria, Jamaica, Mexico, United States, Zambia); others prohibit any form of corporal punishment (e.g., Japan, Mongolia, Scotland, South Africa, New Zealand) (Global Partnership to End Violence Against Children, 2021; ISPCAN, 2021).

In terms of measurement, there are many parental self-report instruments assessing maltreatment (Yoon et al., 2020), and their content appears to differ considerably. This might seem surprising considering that definitions of child maltreatment seem fairly consistent. However, some instruments focus on all aspects of maltreatment, while others focus on specific subtypes (Mathews et al., 2020). But even among instruments of specific subtypes of maltreatment, differences appear to be substantial. This may be because instruments have been developed for different populations (e.g., population sample vs. clinical subsample), and for different purposes (e.g., child protective services vs. research). Also, despite the availability of instruments directly asking about maltreatment, researchers and practitioners often use instruments that measure proxies of maltreatment, such as the Child Abuse Potential Inventory (Milner, 2004) or other inventories asking about risk factors for child maltreatment, for example, parent mental health or financial insecurity (Chen & Chan, 2016; Hood et al., 2021; Reid & Snyder, 2021).

### Defining and Measuring Harsh Parenting

Compared to child maltreatment, definitions of harsh parenting are much less consistent. First, there are studies that do not define harsh parenting and use either the operationalization of their instruments as a way to describe the parenting behaviors they label as harsh parenting (e.g., Choi & Becher, 2019; Cortes Hidalgo et al., 2021; Oshri et al., 2020), or examples instead of a comprehensive definition, such as spanking, slapping, yelling, or shouting (e.g., Jackson & Choi, 2018; Thartori et al., 2019).

Second, there are studies that define harsh parenting in terms of parental attempts to control the child’s behaviors or parent–child relationship by use of anger, coercion, aggression, or emotional reactions (e.g., Eltanamly et al., 2019; Park & Johnston, 2020; Rueger et al., 2011). Most definitions typically include both physical and psychological behaviors. For example, Pinquart (2017) defines harsh control as “physical or verbal punishment and intrusiveness” (p. 873) and Kelly et al. (2021) identify harsh parenting practice as any parental psychological and physical aggression.

Third, there are studies that define harsh parenting using terminology from child maltreatment definitions. These studies then either differentiate harsh parenting from child maltreatment or approach it as the same construct. For example, Evans et al. (2012) define harsh parenting as emotional abuse and physical punishment, where the term “abuse” suggests overlap with maltreatment. In contrast, Berthelon et al. (2020) defined harsh parenting as “parental strategies that incorporate these lesser forms of violence and aggression towards children,” with more severe forms of violence being classified as child maltreatment (p. 2).

In terms of measurement, harsh parenting is often assessed with subscales of instruments designed to measure multiple aspects of parenting (e.g., warmth, behavioral control). Consequently, available systematic reviews of parental self-report instruments on parenting behaviors typically focus on parenting more generally, rather than on harsh parenting specifically (Blower et al., 2019; Hurley et al., 2014; Rodriguez et al., 2021). To our knowledge, no reviews focusing on instruments explicitly designed to measure harsh parenting exist.

### Child Maltreatment and Harsh Parenting in Context

Since harsh parenting is sometimes seen as a milder form of violence it is often not prohibited. In many countries, harsh parenting behaviors such as corporal punishment are the norm for children, supported by cultural traditions and lack, or poor implementation, of legislation (UNICEF, 2014b). However, in past decades, research and global advocacy have shifted toward protecting children from any form of violence, including violence supported by cultural traditions. Corporal punishment and other forms of harsh parenting (e.g., repeated belittling) are now banned in many countries (ISPCAN, 2021). This shift is in line with research showing that harsh parenting behaviors can have similar detrimental consequences to children’s health and development as child maltreatment (Cortes Hidalgo et al., 2021; Gershoff et al., 2018; Gilbert et al., 2009).

Strategies to prevent harsh parenting and maltreatment overlap. For example, the WHO and the UNICEF’s global strategy to end violence against children promotes support to parents and caregivers as a key strategy to prevent violent parenting (WHO, 2016). In practice, the same parenting interventions are often used to reduce harsh parenting and child maltreatment. For example, the 1-2-3 Magic Parenting Program has been separately implemented to reduce harsh parenting (Bailey et al., 2015; Bradley et al., 2003) or child maltreatment (Flaherty & Cooper, 2010), but the contents of the program (e.g., the skills taught to parents) were the same. The same holds for programs such as Triple P Positive Parenting (for harsh parenting: Eisner et al., 2012; for child maltreatment: Lanier et al., 2018); and Incredible Years (for harsh parenting: Azevedo et al., 2014; for child maltreatment: Karjalainen et al., 2019).

The conclusions we draw from research findings, and our approaches to prevention, rely on consistent definitions and operationalizations. If child maltreatment and harsh parenting are meaningfully different constructs, but operationalized similarly in self-report instruments, there is a substantial risk that previous research has overestimated similarities in the consequences of harsh parenting, maltreatment, and prevention efforts. This is because instruments would not have differentiated between the two constructs. If, in contrast, an overlap in instruments designed to measure either child maltreatment or harsh parenting reflects a true overlap in the constructs, previous research in either field (child maltreatment or harsh parenting) may have ignored a large body of evidence when estimating prevalence rates, consequences, and prevention effects due to dissimilar terminology used in different fields for the same constructs. This highlights the need for understanding how much overlap there is in the content of instruments designed to measure either child maltreatment or harsh parenting, and identify where, if at all, instruments include parenting behaviors unique to each construct. In addition, instruments within each category (i.e., child maltreatment and harsh parenting) may be heterogeneous in the parenting behaviors they measure—instruments rarely include all symptoms or behaviors that define a construct (Fried, 2017; Visontay et al., 2019).

The present study, therefore, aimed to unpack (a) whether harsh parenting and child maltreatment instruments measure similar or different parenting behaviors, and (b) how homogeneously instruments include the same parenting behaviors. The specific research questions were:

1. Which and how many physical and emotional parenting behaviors are measured in both harsh parenting and child maltreatment parent self-report instruments?

a. Which parenting behaviors are unique to child maltreatment instruments?
b. Which parenting behaviors are unique to harsh parenting instruments?

2. How homogeneously are parenting behaviors measured in the instruments?
a. How homogeneous are child maltreatment instruments in the behaviors they measure?
b. How homogeneous are harsh parenting instruments in the behaviors they measure?
c. How does the homogeneity across child maltreatment and harsh parenting instruments compare to the homogeneity within each instrument category?

## Methods

### Instrument Selection

**Figure 1.**
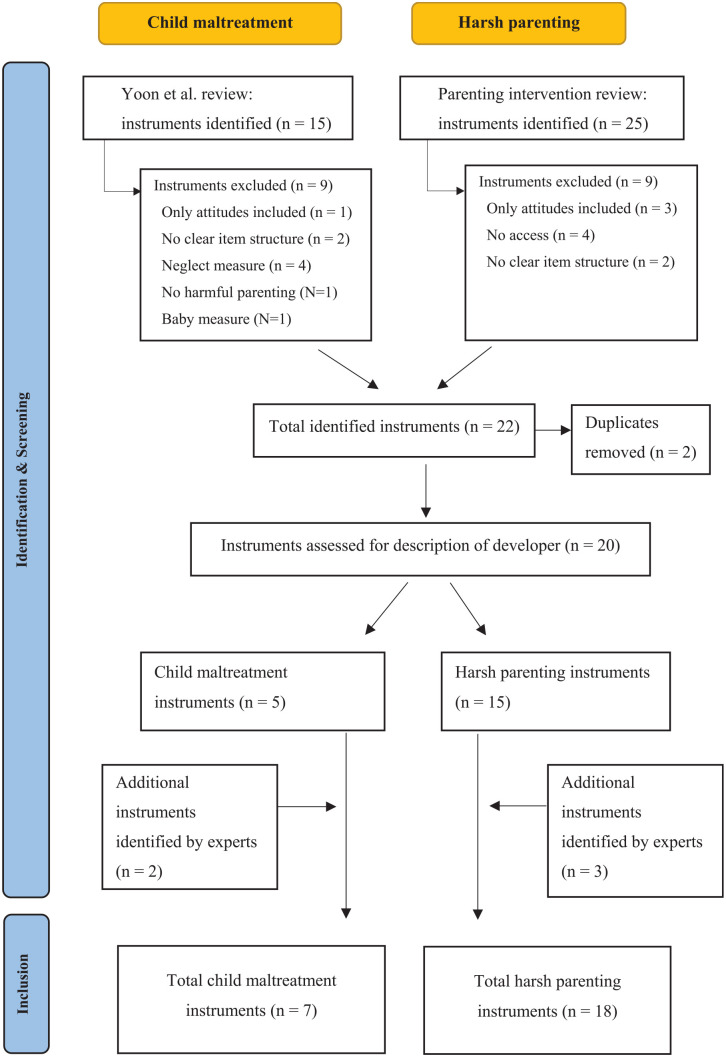
Flowchart reflecting instrument selection.

We aimed to identify parent self-report child maltreatment and harsh parenting instruments that measure physical or emotional harmful parenting. For this, we used two recent systematic reviews, described in detail below. In addition, we contacted 22 leading experts with a track record with publications on parenting and child maltreatment, in the fields of psychology, psychiatry, law, social work, and global and public health, and asked them to add to our list of instruments any additional frequently used instruments (Figure 1).

We coded instruments as measuring child maltreatment or harsh parenting based on descriptions by the instruments’ developers. Instruments were coded as child maltreatment if the developer used terms such as abuse, neglect, or child maltreatment. For example, the Conflict Tactics Scale Parent–Child is often used to measure both harsh parenting and maltreatment, but the developers Straus et al. (1998) described this instrument as a child maltreatment instrument. We, therefore, coded it as measuring child maltreatment.

When two instruments shared the same subscale or items, we treated these instruments as one (to not overestimate similarity between instruments). This applied to the Demographic and Health Surveys (DHS) and the Multiple Clusters Indicator Survey (MICS) that shared the “Child Discipline” module and used the exact same items. Similarly, we merged the ISPCAN Child Abuse Screening Tool (ICAST) and ICAST—Trial Parent versions, since both are administered to parents and are based on each other.

Exclusion criteria for instruments were (a) not measuring physical or emotional harmful parenting behaviors (e.g., neglect only), (b) not a parent self-report instrument, (c) measuring attitudes or risk for maltreatment but not behaviors, (d) instrument having a different item structure (e.g., timing for a video, semi-structured interview), (e) instrument was unavailable, and (f) instrument focused exclusively on abusive head trauma.

### Child Maltreatment Instruments

We identified child maltreatment instruments using the recent systematic review by Yoon et al. (2020), who searched six electronic databases in October 2019 and identified 15 parent self-report instruments. Of these, six instruments were included in the present study. We excluded the following instruments: (a) four that focused only on neglect (Child Neglect Questionnaire, Child Neglect Scales–Maternal Monitoring and Supervision scale, the Mother–Child Neglect Scale, the Mother–Child Neglect Scale–Short Form); (b) one that did not measure any harmful parenting behaviors (Parent Opinion Questionnaire); (c) two that did not have a clear item structure, but rather asked respondents to stop a movie clip when they judged a scene to have become abusive (Analog Parenting Task, Parent–Child Aggression Acceptability Movie Task); (d) one that measured attitudes toward maltreatment rather than actual parenting behaviors (Adult Adolescent Parenting Inventory-2); and (e) one that assessed abusive head trauma (Shaken Baby Syndrome awareness assessment–Short Version; see Online Appendix A for list of excluded studies).

### Harsh Parenting Instruments

We identified harsh parenting instruments using an ongoing global systematic review of the effects of parenting interventions to reduce harsh parenting and child maltreatment (PROSPERO registration CRD42019141844). This review used extensive searching in 12 English and 14 non-English databases and platforms in multiple languages (English, Spanish, Chinese, Farsi, Russian, Thai), and an exhaustive grey literature search; all searches were conducted in August 2019. Search terms surrounded three conceptual categories: (a) intervention, (b) parenting, and (c) child behavioral and emotional problems or maltreatment/violence. More information on the search can be found in the online protocol of this review. We identified a total of 278 trials of which 98 measured harsh parenting with 25 unique instruments. We included parent self-report instruments that measured harsh parenting or included a subscale of a general parenting inventory that focused on harsh parenting. Even though we prefer to avoid posing a new definition, for transparency, we defined harsh parenting for the purpose of selecting instruments from this review as *physical or verbal punishment or aggression, words or acts that cause harm, potential harm or threat of harm to a child.* A total of 18 instruments met this description (see Table 2). We excluded 10 instruments because (a) two instruments used open-ended questions rather than asking about specific parenting behavior (Harsh Discipline scale, Parent Daily Telephone Reports); (b) three instruments only included attitudes (Adult Adolescent Parenting Inventory-2, Child Abuse Potential Inventory, Parent’s Attributions to Child’s Behavior Measure); (c) one instrument used mainly observation (HOME–Short Form); and (d) four instruments were not accessible (Angry Outbursts scale, Harsh Punishment scale, Ideas about Parenting, Nijmegen Parenting Questionnaire).

### Overall Included Instruments

We included 7 instruments to measure child maltreatment and 18 instruments to measure behaviors that were grouped under harsh parenting (Tables 1 and 2). Because we coded instruments based on what they were originally developed to measure, two instruments identified by Yoon et al. (2020) were coded as harsh parenting: Intensity of Parental Punishment Scale, and Parental Response to Child Misbehavior. Experts (response rate: 50%) recommended the inclusion of two additional child maltreatment instruments (Comprehensive Child Maltreatment Scale & Juvenile Victimization Questionnaire–Parent Version) and three harsh parenting instruments (Dimensions of Discipline Inventory; Parental Acceptance–Rejection Questionnaire; Parenting Styles and Dimensions Questionnaire). Most harsh parenting instruments were part of a more general parenting inventory that included subscales on harsh parenting. Various child maltreatment instruments measured multiple forms of child maltreatment including neglect and sexual abuse. These items were excluded from this review, even if the instrument itself was included, because neglect and sexual abuse clearly meet the definition for child maltreatment only and are not considered in measures of harsh parenting. The majority of included instruments were developed in the 1990s (median year of development: 1999). Included child maltreatment instruments were developed between 1998 and 2019 (median: 2005); harsh parenting instruments were generally somewhat older and developed between 1975 and 2014 (median: 1995).

**Table 1.** Included Instruments Measuring Child Maltreatment.

Abb.	Instrument Full Name	Developer	Subscales	Items Total	% Included Items—Total Scale	% Included Items—Subscale
CCMS	Comprehensive Child Maltreatment Scale	Higgins and McCabe (2001)	5	22	82	100
CTS-ES	Child Trauma Screen–Exposure Score	Lang and Connell (2017)	1	4	40	100 (Potentially traumatic exposure)
CTSPC	Conflict Tactics Scale Parent–Child	Straus et al. (1998)	4	27	67	100 (Physical assault); 100 (psychological aggression)
DHS/MICS	Child discipline module shared by DHS and MICS surveys	DHS, MICS	—	12	67	*No subscale*
FM-CA	Family Maltreatment- Child Abuse criteria	Heyman et al. (2020)	2	28	100	100 (Physical abuse); 100 (emotional abuse)
ICAST-P/-T	ISPCAN Screening Tool for use in Trials & Parent-version (combined)	Meinck et al. (2018); Runyan et al. (2009)	4	14	65	100 (Physical discipline); 100 (severe physical discipline); 100 (psychological discipline)
JVQ	Juvenile Victimization Questionnaire	Finkelhor et al. (2005)	4	20	6	40 (Child maltreatment)

**Table 2.** Included Instruments Measuring Harsh Parenting.

Abb.	Instrument Full Name	Developer	Subscales	Items Total	% Included Items—Total Scale	% Included Items—Subscale
APQ	Alabama Parenting Questionnaire–Parent Form	Frick (1991)	6	42	10	14 (Other disciplining); 100 (corporal punishment)
BSI	Brief Symptom Inventory	Derogatis (1983)	9	53	9	100 (Hostility)
CCNES	Coping With Child Negative Emotions	Fabes (1990)	6	72	17	100 (Punitive reactions)
DDI	Dimensions of Discipline Inventory (Part C)	Straus and Fauchier (2007)	9	26	27	100 (Corporal punishment); 75 (psychological aggression)
GPBS	Ghent Parental Behavior Scale	Van Leeuwen and Vermulst (2004)	9	45	8	100 (Harsh punishment)
HDPL	Harsh Discipline Practice List	Jiménez Flores and Flores Herrera (2014)	2	19	100	*No subscale*
HS	Harsh Scale	Miller-Heyl et al. (1998)	2	11	36	66 (Harsh punishment)
IPPS	Intensity of Parental Punishment Scale	Gordon et al. (1979)	5	33	100	*No subscale*
PAFAS	Parenting and Family Adjustment Scale	Sanders et al. (2014)	9	30	10	60 (Coercive parenting)
PARQ	Parental Acceptance–Rejection Questionnaire	Rohner et al. (2005)	4	60	23	60 (Hostility/aggression); 50 (rejection)
PBC	Parent Behavior Checklist	Fox (1994); Brenner and Fox (1998)	3	100	21	70 (Discipline: verbal and corporal punishment)
PBI	Parent Behavior Inventory	Lovejoy et al. (1999)	2	20	25	50 (Hostile/coercive)
PPI	Parent Practice Interview	Webster-Stratton (1998)	6	73	12	100 (Physical punishment); 27 (harsh and inconsistent discipline)
PPS	Parenting Practice Scale	Strayhorn and Weidman (1988)	0	34	18	*No subscale*
PQ	Parenting Questionnaire	McCabe and Clark (1999)	3	50	12	5 (Warmth); 100 (corporal punishment)
PRCM	Parental Response to Child Misbehavior Questionnaire	Holden et al. (1995)	0	12	50	*No subscale*
PS	Parenting Scale	Arnold et al. (1993)	3	32	13	40 (Overreactivity)
PSDQ	Parenting Styles and Dimensions Questionnaire	Robinson et al. (1995)	3	62	13	40 (Authoritarian items)

### Thematic Analysis to Identify Parenting Behaviors

We conducted a qualitative thematic analysis of items across all instruments (Braun & Clarke, 2006; Vaismoradi et al., 2013). This method allows us to identify and analyze the text of the items and translate the content of items to concrete behaviors that describe harmful parenting behaviors. We followed a deductive approach for identifying and including physical and emotional harmful parenting behaviors as initial coding themes from the data corpus into our dataset (Hsieh & Shannon, 2005). Our initial definition of harsh parenting served as a framework to identify items measuring physical and emotional harmful parenting behaviors.

We used the phases of thematic analysis as outlined by Braun and Clarke (2006). First, we reviewed and entered all eligible items into a spreadsheet. Second, we developed initial codes and grouped items under physical or emotional harmful behaviors. Third, we developed themes for specific parenting behaviors in a deductive and indicative coding approach. For example, “Spanked him or her on the bottom with your bare hand,” “I spank my child at least once a week,” and “I hit or spanked child using a stick, hairbrush, or some other hand object” were grouped under the specific parenting behavior theme *spank or hit with object or hand* in the overarching category of *physical behaviors*. Some items were placed under multiple behaviors since they described a multitude of parenting behaviors. For example, “I spank, grab, slap, or hit my child most of the time” was grouped in physical behaviors, and then placed under three parenting behavior themes (“Spank or hit with object or hand,” “Grab,” and “Slap”). Fourth, we reviewed the developed themes, and, fifth, defined, merged, and named themes.

All instruments combined included 949 items. Of these, 243 items (104 from child maltreatment; 139 from harsh parenting instruments) reflected potentially harmful physical or emotional parenting behaviors. We grouped these 243 eligible items under 22 thematic clusters of parenting behaviors (Table 3), including 17 physical and 5 emotional parenting behaviors.

**Table 3.** Prevalence of Occurrence of Thematic Clusters Identified in Harsh Parenting and Child Maltreatment Instruments.

	Child Maltreatment			Harsh Parenting		
Physical behaviors	0%	50%	100%	0%	50%	100%
	Spank/hit with object or hand 86% Slap 43% Grab 29% Hit hard/hit with fist/beat up 100% Shake 71% Hit with knuckles 14% Give harmful food/liquid 14% Kick 71% Pinch 43% Pull hair 14% Push/throw down 29% Burn 43% Choke 43% Force to stand or kneel 29% Tie up 29% Twist 29% Withhold meal 14%			Spank/hit with object or hand 89% Slap 44% Grab 22% Hit hard/hit with fist/beat up 11% Shake 11% Hit with knuckles 6% Give harmful food/liquid 6% Kick 6% Pinch 6% Pull hair 6% Push/throw down 6% Burn 0% Choke 0% Force to stand or kneel 0% Tie up 0% Twist 0% Withhold meal 0%		
Emotional behaviors	0%	50%	100%	0%	50%	100%
	Scold/yell/shout/scream 43% Threaten 57% Insult/humiliate/call names 86% Blaming/making fun of 14% Curse/swear 29%			Scold/yell/shout/scream 72% Threaten 44% Insult/humiliate/call names 28% Blaming/making fun of 17% Curse/swear 6%		

Given the purpose of the study to identify the overlap in harsh parenting and child maltreatment, items measuring other forms of parenting, such as overprotection, were not considered a relevant theme and removed from the analysis. We further excluded items if no clear indication of potential harm was given. For example, criticizing children may have strong negative effects, for example if children are constantly criticized without being praised (Gunderson et al., 2018). However, constructive criticism may not hamper but support developmental growth of children. Therefore, items that were not clearly potentially harmful, were omitted (for example, “I use criticism to improve my child”). In addition, we excluded items that did not clearly reflect a concrete parenting behavior (for example, “I am harsh with my child,” “I punish my child”), and behaviors that were not clearly directed at the child (e.g., “I complain about my child” or “I wonder if I really love my child”).

### Estimating Similarity Between Child Maltreatment and Harsh Parenting Instruments

We listed all behaviors included in child maltreatment instruments and in harsh parenting instruments, and listed (a) which behaviors were identified in both instrument categories, (b) which behaviors were unique to child maltreatment instruments, and (c) which behaviors were unique to harsh parenting instruments. We then calculated the overall percentage of overlap between child maltreatment and harsh parenting instruments by taking the number of behaviors that were identified in both harsh parenting and child maltreatment instruments, and dividing it by the total number of physical and emotional parenting behaviors we identified in our thematic analysis.

### Comparing Homogeneity Across and Within Instrument Categories

We expected that not all instruments included the exact same parenting behaviors, as also indicated by the differing length of instruments. To quantify these varying levels of homogeneity between instruments, we calculated the Jaccard Index (Chrobak et al., 2018; Fried, 2017; Visontay et al., 2019; Wall & Lee, 2021), a measure of the similarity between two sets of binary data. The Jaccard coefficient ranges from 0 (*fully heterogeneous set of instruments*) to 1 (*fully homogeneous set of instruments*). It is calculated for each pairwise combination of instruments by taking the number of shared parenting behaviors divided by the total number of parenting behaviors across both instruments (shared and unique parenting behaviors). The analysis can be expressed as *s*/(*u*1 + *u*2 + *s*), where *s* represents the number of behaviors two instruments share, and *u*1 and *u*2 the number of behaviors that are unique to each of the two instruments (Fried, 2017). We used Fried’s criteria whereby a very weak Jaccard Index is 0.00 to 0.19, weak 0.20 to 0.39, moderate 0.40 to 0.59, strong 0.60 to 0.79, and very strong 0.80 to 1.00 (proposed by Fried, 2017, based on Evans, 1996). Analyses were conducted in R Statistics using adjusted code supplied by Fried (2017).

We ran three sets of analyses. First and second, we analyzed the homogeneity within child maltreatment instruments and within harsh parenting instruments. For each instrument category, we calculated (1) the overall homogeneity across all parenting behaviors, (2) the homogeneity in physical behaviors specifically, and (3) the homogeneity in emotional behaviors specifically. Third, we analyzed how homogeneous harsh parenting instruments are with child maltreatment instruments. For this, we compared the share of items of each harsh parenting instrument to each child maltreatment instrument. In addition, we calculated the levels of homogeneity separately for physical and emotional parenting behaviors.

## Results

### Overall Similarity Across Child Maltreatment and Harsh Parenting Instruments

We observed considerable overlap in parenting behaviors measured across harsh parenting and child maltreatment instruments. Of the 22 parenting behaviors (Table 3), 16 were shared between both instruments (73%). Moreover, 100% of the parenting behaviors included in harsh parenting instruments were also included in child maltreatment instruments. This indicates that most of the content for child maltreatment and harsh parenting instruments is shared rather than being unique.

### Similarity in Physical Parenting Behaviors

The instruments included 17 physical behaviors. Some behaviors were more prevalent in harsh parenting instruments, whereas some more often included in child maltreatment instruments. Most prevalent behaviors included in child maltreatment instruments were “hit hard or hit with a fist, or beat up,” and “spank or hit with the hand or object” (see Tables 3 and 4). Most instruments also measured kicking and shaking. Less common were items on slapping, pinching, choking, burning, grabbing, throwing down or pushing, tying up, and twisting. Some behaviors were only measured by one instrument: hit with knuckles, pull hair, give child harmful food or liquid, and withhold a meal.

**Table 4.** Physical Behaviors Included in Each Instrument.

		P1	P2	P3	P4	P5	P6	P7	P8	P9	P10	P11	P12	P13	P14	P15	P16	P17
APQ	Harsh parenting	X	X															
BSI	Harsh parenting				X													
CCNES	Harsh parenting																	
CCMS	Child maltreatment	X		X	X		X			X								
CTS-ES	Child maltreatment				X		X											
CTSPC	Child maltreatment	X	X		X			X		X	X	X			X			
DDI	Harsh parenting	X	X	X						X				X				
DHS/MICS	Child maltreatment	X			X					X								
FM-CA	Child maltreatment	X	X	X	X		X	X		X	X	X	X		X	X	X	
GPBS	Harsh parenting	X	X							X								
HDPL	Harsh parenting	X			X	X	X	X	X									
HS	Harsh parenting	X																
ICAST-P/-T	Child maltreatment	X	X		X	X	X	X	X	X	X	X	X	X		X	X	X
IPPS	Harsh parenting	X																
JVQ	Child maltreatment	X			X		X											
PAFAS	Harsh parenting	X																
PARQ	Harsh parenting	X																
PBC	Harsh parenting	X	X															
PBI	Harsh parenting	X		X														
PPI	Harsh parenting	X	X															
PPS	Harsh parenting	X																
PQ	Harsh parenting	X	X															
PRCM	Harsh parenting	X	X															
PS	Harsh parenting	X		X														
PSDQ	Harsh parenting	X	X	X											X			

*Note*. P1 = Spank/hit with object or hand; P2 = Slap; P3 = Grab; P4 = Hit hard/Hit with first/Beat up; P5 = Hit with knuckles; P6 = Kick; P7 = Pinch; P8 = Pull hair; P9 = Shake; P10 = Burn; P11 = Choke; P12 = Forced to stand or kneel; P13 = Give harmful food or liquid; P14 = Push/Shove/Throw Down; P15 = Tie up; P16 = Twist; P17 = Withhold meal. ; APQ = Alabama Parenting Questionnaire; DHS = Demographic Health Survey; MICS = Multiple Clusters Indicator Survey; ICAST = ISPCAN Child Abuse Screening Tool; CTS-ES = Child Trauma Screen–Exposure Score; CCMS = Comprehensive Child Maltreatment Scale; PAFAS = Parenting and Family Adjustment Scale; HDPL = Harsh Discipline Practice List; JVQ = Juvenile Victimization Questionnaire; PBI = Parent Behavior Inventory; FM-CA = Family Maltreatment–Child Abuse criteria; BSI = Brief Symptom Inventory; CCNES = Coping With Child Negative Emotions; CTSPC = Conflict Tactics Scale Parent–Child; GPBS = Ghent Parental Behavior Scale; HS = Harsh Scale; IPPS = Intensity of Parental Punishment Scale; PARQ = Parental Acceptance–Rejection Questionnaire; PBC = Parent Behavior Checklist; PPI = Parent Practice Interview; PPS = Parenting Practice Scale; PQ = Parenting Questionnaire; PRCM = Parental Response to Child Misbehavior Questionnaire; PS = Parenting Scale; PSDQ = Parenting Styles and Dimensions Questionnaire.

Spank or hit with object or hand was also one of the most frequently included physical behaviors in harsh parenting instruments, and, also similar to child maltreatment instruments, 44% included slapping a child (Tables 3 and 4). Some harsh parenting instruments included grabbing, shaking, hitting hard or hit with fist or beat up, whereas only one instrument measured hit with knuckles, kick, pinch, pull hair, push or throw down, and give harmful food or liquid.

All eleven behaviors included in harsh parenting instruments were also included in child maltreatment instruments: (a) spank or hit child with object or hand, (b) slap, (c) pinch, (d) kick, (e) pull hair, (f) shake, (g) grab, (h) hit hard or hit with fist or beat up, (i) push or throw down, (j) give harmful liquids or food (e.g., spicy food, soap, or alcohol), and (k) hit with knuckles. Six parenting behaviors were only included in child maltreatment instruments: (a) burn, (b) choke, (c) force a child to stand or kneel, (d) tie up, (e) twist a body part, and (f) withhold a meal. None of the behaviors identified only in child maltreatment instruments were included in the majority of child maltreatment instruments (range 14−43%)

### Similarity in Emotional Parenting Behaviors

The instruments included five emotional behaviors. All emotional behaviors that we identified in our thematic analysis were included in both harsh parenting and child maltreatment instruments: scold or yell or shout or scream, threaten, insult or humiliate, blame or make fun of, and curse or swear. Most child maltreatment instruments asked parents whether they insult, humiliate, or call their child names, followed by threatening a child (see Tables 3 and 5). Less prevalent were scolding or yelling or shouting or screaming, cursing or swearing, and blaming or making fun of a child. In contrast, most harsh parenting instruments included items on scolding, yelling, shouting, or screaming. Less prevalent behaviors were threatening, insulting, humiliating, calling names, blaming or making fun of the child, and cursing or swearing.

### Homogeneity in Instrument Content Across and Within Instrument Categories

#### Homogeneity Within Child Maltreatment Instruments

Child maltreatment instruments shared a weak Jaccard Index of 0.35 indicating on average low homogeneity in the included parenting behaviors. Levels of homogeneity between child maltreatment instruments ranged from 8 to 59%, indicating that no child maltreatment instrument included the same behaviors (see Online Appendix B, Figure 3a), but instead that for the most part, different physical and emotional behaviors were measured between child maltreatment instruments.

In terms of physical parenting behaviors specifically, the Jaccard Index was similar (0.36) to the overall comparison. This indicates that maltreatment instruments differed strongly in which physical behaviors they included with only a few behaviors shared between instruments. Levels of homogeneity in physical behaviors between child maltreatment instruments ranged from 11 to 67% (see Online Appendix B, Figure 3b).

In terms of emotional parenting behaviors specifically, we found a higher Jaccard index of 0.47 across child maltreatment instruments. Child maltreatment instruments were thus more homogenous with regard to the emotional parenting behaviors they included compared to the physical behaviors: various instruments were very different in terms of physical behaviors, but shared similar emotional parenting behaviors (e.g., Comprehensive Child Maltreatment Scale [CCMS] and Child Trauma Screen–Exposure Score [CTS-ES]; see Online Appendix B). Homogeneity in emotional behaviors between child maltreatment instruments ranged from 20 to 100% (see Online Appendix B, Figure 3c).

#### Homogeneity Within Harsh Parenting Instruments

Harsh parenting instruments shared a weak Jaccard Index of 0.33, indicating on average low homogeneity in the included parenting behaviors. Homogeneity within harsh parenting instruments ranged from 0 to 100% indicating that some instruments could be used interchangeably, whereas other instruments measured completely different behaviors (see Online Appendix B, Figure 4a).

In terms of physical parenting behaviors specifically, the Jaccard Index was higher (0.42), indicating that harsh parenting instruments were moderately homogenous in which physical parenting behaviors they included. Homogeneity in physical behaviors ranged from 0 to 100% (see Online Appendix B, Figure 4b).

In terms of emotional parenting behaviors specifically, we found a similarly moderate Jaccard Index of 0.47 across harsh parenting instruments. This indicates that harsh parenting instruments were moderately homogenous in the emotional behaviors they included. Homogeneity in emotional behaviors ranged from 0 to 100% (see Online Appendix B, Figure 4c).

#### Homogeneity Between Harsh Parenting and Child Maltreatment Instruments

We found similar weak levels of homogeneity comparing harsh parenting and child maltreatment instruments (Jaccard Index = 0.20), in terms of all behaviors across all behavior categories. Some child maltreatment and harsh parenting instruments, however, were more homogeneous (see Tables 4 and 5). For example, the DHS/MICS Child Discipline Module parental report (child maltreatment) and Parenting and Family Adjustment Scales (PAFAS) (harsh parenting) included 60% of the same physical and emotional parenting behaviors. Some instruments showed higher levels of homogeneity with instruments from the other category than to instruments from their own: the Harsh Discipline Practice List (HDPL), a harsh parenting instrument, shared on average 39% of the same parenting behaviors with child maltreatment instruments (average Jaccard = 0.39) and only 23% with other harsh parenting instruments (average Jaccard = 0.23). However, most harsh parenting and child maltreatment instruments showed very weak or weak levels of homogeneity (e.g., Alabama Parenting Questionnaire and CTS-ES = 0%; see Online Appendix B, Figure 2a).

**Table 5.** Emotional Behaviors Included in Each Instrument.

		E1	E2	E3	E4	E5
APQ	Harsh parenting	X				
BSI	Harsh parenting					
CCNES	Harsh parenting	X				
CCMS	Child maltreatment		X	X		
CTS-ES	Child maltreatment					
CTSPC	Child maltreatment	X	X	X		X
DHS/MICS	Child maltreatment	X		X		
FM-CA	Child maltreatment		X	X		
GPBS	Harsh parenting					
HDPL	Harsh parenting	X	X	X		
HS	Harsh parenting	X	X			
ICAST-P/-T	Child maltreatment	X	X	X	X	X
IPPS	Harsh parenting					
JVQ	Child maltreatment			X		
PAFAS	Harsh parenting	X		X		
PARQ	Harsh parenting	X	X	X	X	
PBC	Harsh parenting	X			X	
PBI	Harsh parenting		X	X		
PPI	Harsh parenting	X				
PPS	Harsh parenting	X	X		X	
PQ	Harsh parenting	X	X			
PRCM	Harsh parenting	X	X			
PS	Harsh parenting	X		X		X
PSDQ	Harsh parenting	X	X			

*Note*. E1 = Scold/yell/shout/scream; E2 = Threaten; E3 = Insult/humiliate/call names; E4 = Blame/make fun of; E5 = Curse/swear; APQ = Alabama Parenting Questionnaire; DHS = Demographic Health Survey; MICS = Multiple Clusters Indicator Survey; ICAST = ISPCAN Child Abuse Screening Tool; CTS-ES = Child Trauma Screen–Exposure Score; CCMS = Comprehensive Child Maltreatment Scale; PAFAS = Parenting and Family Adjustment Scale; HDPL = Harsh Discipline Practice List; JVQ = Juvenile Victimization Questionnaire; PBI = Parent Behavior Inventory; FM-CA = Family Maltreatment–Child Abuse criteria; BSI = Brief Symptom Inventory; CCNES = Coping With Child Negative Emotions; CTSPC = Conflict Tactics Scale Parent–Child; GPBS = Ghent Parental Behavior Scale; HS = Harsh Scale; IPPS = Intensity of Parental Punishment Scale; PARQ = Parental Acceptance–Rejection Questionnaire; PBC = Parent Behavior Checklist; PPI = Parent Practice Interview; PPS = Parenting Practice Scale; PQ = Parenting Questionnaire; PRCM = Parental Response to Child Misbehavior Questionnaire; PS = Parenting Scale; PSDQ = Parenting Styles and Dimensions Questionnaire.

For physical behaviors only, we found a very weak Jaccard Index of 0.19 between harsh parenting and child maltreatment instruments, indicating that harsh parenting and child maltreatment instruments are similarly heterogeneous regarding which physical parenting behaviors they include (see Online Appendix B, Figure 2b). However, there were some exceptions (e.g., HDPL and Juvenile Victimization Questionnaire; Jaccard Index = 0.50).

For emotional behaviors only, the Jaccard Index increased to a weak index of 0.36, indicating higher levels of shared emotional parenting behaviors between harsh parenting and child maltreatment instruments (Figure 2c). There were multiple child maltreatment and harsh parenting instruments that showed complete homogeneity (Jaccard Index = 1.00; Parent Behavior Inventory [PBI] and CCMS, PBI and Family Maltreatment–Child Abuse criteria, and DHS/MICS Child discipline and PAFAS), indicating that these instruments included exactly the same emotional parenting behaviors. In fact, several harsh parenting instruments (e.g., HDPL and PBI) showed more homogeneity with child maltreatment instruments than with other harsh parenting instruments.

## Discussion

How we measure constructs in violence research impacts our research findings, policy discussions, and prevention approaches. This study compared the parenting behaviors included in parent self-report instruments developed to measure child maltreatment and instruments developed to measure harsh parenting. We found that 73% of parenting behaviors were included in both child maltreatment and harsh parenting instruments, suggesting substantial but not complete similarity. Various behaviors were only included in child maltreatment instruments such as burning and withholding a meal.

Our findings raise the question of whether the parenting behaviors uniquely included in maltreatment instruments are indeed the behaviors that distinguish maltreating families from non-maltreating, harsh parenting families. On the one hand, some of these behaviors, such as burning or choking, might indeed reflect particularly harmful parenting behaviors with severe consequences (Manly et al., 2001). On the other hand, for many behaviors, it may be difficult to judge how severe they are relative to other behaviors. For example, twisting a body part is unique to child maltreatment instruments and may or may not be more severe than pulling hair, which can also be found in harsh parenting instruments. Because some scholars suggest that harsh parenting is a mild form of child maltreatment (e.g., Berthelon et al., 2020), it is important to know to what extent the behaviors uniquely included in child maltreatment instruments indeed indicate more harmful behaviors. Moreover, apart from severity, studies indicate that the age of the child at the time of the violent events, as well as whether children experience multiple types of abuse, determine the impact of these events on children’s developmental outcomes (Hanlon et al., 2020; Manly et al., 2002).

The behaviors unique to child maltreatment instruments may also indicate less prevalent forms of violence, rather than necessarily less or more severe parenting behaviors. In the absence of a review of the most prevalent harmful parenting behaviors, we turn to examples of individual studies on behavior frequency and severity. The developers of the ICAST-P identified that moderate physical discipline was nearly 5 times as prevalent across six countries than severe physical discipline (Runyan et al., 2009). Less than 10% of respondents indicated that they burned or choked their child, forced their child to stand or kneel, gave hot chili pepper to the child, or withheld a meal as a punishment. More frequently reported were spanking (37%), shaking a child (30%), or pinching and slapping (25%), all of which are also included in harsh parenting instruments. Similarly, Ismayilova and Karimli (2020) found that parents frequently reported spanking, hitting or slapping (68%), shaking (53%), or hitting on the face, head, or ears (54%); whereas children reported most frequently experiences of hitting, beating, or spanking with a belt, stick, or other object (29%) followed by pulling hair, pinching roughly, and twisting ear (23%), and pushing, kicking, grabbing, shoving, slapping, or throwing something at them (14%). All of these most prevalent behaviors can be found in harsh parenting instruments. Very few children reported being choked or burned, or having experienced parental attempts to drown them (2%). Even though these are just two studies, they suggest that the unique child maltreatment behaviors measure to some extent severe but rare forms of violent parenting behaviors, and that the most prominent behaviors are included in both child maltreatment and harsh parenting instruments. If this were true, then maltreating versus not maltreating families might often be distinguished based on scores on items that are not unique to child maltreatment instruments, but that are also included in harsh parenting instruments.

Homogeneity in the parenting behaviors measured between individual instruments, both across and within categories, was generally low. Although some instruments shared all behaviors (Jaccard Index = 1.0), others measured entirely different parenting behaviors (Jaccard Index = 0.00) This is not uncommon for instruments measuring other constructs (e.g., depression, Fried, 2017; anxiety, Wall & Lee, 2021). Some form of heterogeneity may be needed to capture the harmful parenting behaviors dominantly used in different cultural setting (Bent-Goodley, 2021). However, consensus on the most prevalent harmful parenting behaviors that need to be included in instruments is crucial. Otherwise, the consequence of low levels of homogeneity between instruments is that there may be a risk that the selection of a particular instrument can bias the results. For example, prevalence rates or intervention effectiveness may be underestimated if the prominent harmful behaviors used by the reporting parent are not included in the chosen instrument.

That said, average levels of homogeneity were slightly higher within each category than between. This might indicate that researchers within each field might have more consensus on which key parenting behaviors are harsh parenting and which are child maltreatment. However, there may be other explanations. First, instruments are often based on each other within one instrument category which in turn often leads to including the same or similar items. This seems to be the case, for example, with the DHS/MICS Child Discipline module and the ICAST-P, as they are both based on the CTSPC. Second, instruments within categories might be developed more often for the same purpose. Harsh parenting instruments seem to be used more often in longitudinal studies of parenting styles and intervention trials, whereas child maltreatment instruments are often used to record all potential harmful parenting behaviors to estimate prevalence rates. Third, differing length of instruments, and consequently of included parenting behaviors, between harsh parenting (average 10 items measuring four distinct parenting behaviors) and child maltreatment instruments (average 14 items measuring nine behaviors) may impact the homogeneity index. Comparing a shorter harsh parenting instrument to a longer maltreatment instrument yields less shared behavior content. Importantly, however, different lengths might also reflect meaningful differences, with harsh parenting instrument developers agreeing more on what the key parenting behaviors are that reflect their construct.

This study has several strengths. To understand how to protect children from harmful parenting and its implications on child development, a clear consensus on the underlying construct, terminology, and instruments that measure those parenting behaviors is crucial. This study contributes to this need by laying the foundation for discussions on the similarity between harsh parenting and child maltreatment in meaning and measurement. For this, we used state-of-the-art mixed methods, including systematic reviews for instrument selection, qualitative assessment of item content of included instruments, and a quantitative analysis of the overlap and homogeneity of the identified parenting behaviors using the Jaccard Index.

This study also has several limitations. First, although our procedure to identify and include instruments was systematic, it does not include all instruments used in the field. Because of a lack of systematic reviews on harsh parenting instruments, we used a parenting intervention review to identify harsh parenting instruments. As such, we may have missed some harsh parenting instruments that are not used in parenting intervention trials. However, we aimed to account for any important missing harsh parenting instruments that may be prevalent outside of the parenting intervention literature by contacting leading experts from the harsh parenting literature and asking for any missing key instruments for subsequent inclusion. Second, we focused only on physical and emotional parenting behaviors, and not on other forms of child maltreatment (e.g., neglect or sexual abuse). We opted for this approach, because those behaviors are most prevalent and typically associated with harsh parenting and child maltreatment. Also, neglect and sexual abuse are often measured with separate instruments (e.g., Mother Child Neglect Scale and Abusive Sexual Exposure Scale) and clearly defined as maltreatment and therefore do not occur in conceptualizations or measures of harsh parenting. Third, deductive qualitative analysis can lead to a more informed data approach but is also prone to bias (Hsieh & Shannon, 2005). Using a predefined definition of harsh parenting and child maltreatment may have led to finding items that are supportive rather than non-supportive of the definition. We also acknowledge that the 22 identified parenting behaviors could have been categorized differently, such as separating out pushing and throwing down, which could imply different levels of violence severity. However, most items did not specify severity of parenting behavior. Fourth, the parenting behaviors that are classified as harsh, violent, and potentially harmful may vary by culture (ISPCAN, 2021). We acknowledge that our own cultural biases potentially influenced our decision to include some behaviors and not others (Tajima, 2021). Importantly, however, we used a definition to classify behaviors as harsh, and observed considerable overlap in parenting behaviors between instruments, indicating that the behaviors included at least reflect consensus across researchers operating in different fields and countries. Fifth, this study focused on the operationalization of constructs in instruments. We acknowledge the need for a review on the constructs of harsh parenting and child maltreatment, and additional research on the similarity and distinction of the two constructs.

Our findings show that instruments of child maltreatment and harsh parenting substantially share the same parenting behaviors. In fact, any behavior included in harsh parenting instruments is also included in child maltreatment instruments. That said, child maltreatment instruments did include several unique behaviors, such as twisting a body part and burning. Agreement on the parenting behaviors to include in instruments was only slightly higher within each construct category (homogeneity within harsh parenting and within child maltreatment instruments) than between child maltreatment and harsh parenting instruments. These findings have clear implications for research, policy, and practice. For researchers, there is a substantial risk of missing out a large body of literature when estimating the global knowledge of child maltreatment if findings from highly overlapping instruments are ignored on the basis of being labeled as harsh parenting rather than maltreatment. This could have a severe effect, particularly in research synthesis and may lead to under/or overestimates of prevention efforts, associations, and prevalence. For policy, legislation and policies are often informed by research findings. While there seems to be a strong need for consensus in the research field, policy makers need clear terminology that describes the actual behaviors measured in evidence in order to ensure services and provisions are targeted correctly.

For practice, it is crucial to identify which parenting behaviors may or may not distinguish child maltreatment from harsh parenting, as this may affect how families are perceived by services and which interventions may be offered.

Consequently, there is a clear need for a discussion on whether harsh parenting and child maltreatment should be defined and measured as similar or different constructs. One way to shed light on this is by examining the unique versus shared predictive value of scores based on instruments designed to either measure harsh parenting or child maltreatment. This could perhaps extend current important debates, for example on whether spanking should be classified as child maltreatment and an adverse childhood experience (Afifi et al., 2017; Gershoff et al., 2018). Should the field decide that harsh parenting and child maltreatment are more similar than different, terminology optimally would be limited to one term that clearly describes the construct. One option may be UNICEF’s use of “violence against children.” If, in contrast, the field decides that harsh parenting and child maltreatment, even though currently measured similarly, are indeed two meaningfully distinct constructs and should be treated as such, researchers need to invest in developing instruments that clearly distinguish between those constructs, and thus between maltreating and non-maltreating harsh parenting families. Finally, there is a strong need for agreement on which behaviors should be included in instruments to minimize the risks attached to the selection of heterogeneous instruments. We showed that there is substantial overlap in harmful parenting behaviors measured in harsh parenting and child maltreatment instruments. For effective prevention and detection of violence against children, we must further develop conceptualizations and operationalizations of harsh parenting versus maltreatment and ensure that instruments are available to either estimate each construct respectively, or one overarching construct, depending on the consensus decision.

## Supplemental Material

sj-docx-1-tva-10.1177_15248380221134290 – Supplemental material for Different Instruments, Same Content? A Systematic Comparison of Child Maltreatment and Harsh Parenting Instruments Click here for additional data file. Supplemental material, sj-docx-1-tva-10.1177_15248380221134290 for Different Instruments, Same Content? A Systematic Comparison of Child Maltreatment and Harsh Parenting Instruments by Sophia Backhaus, Patty Leijten, Franziska Meinck and Frances Gardner in Trauma, Violence, & Abuse
