# Supplementary material for: Different Instruments, Same Content? A Systematic Comparison of Child Maltreatment and Harsh Parenting Instruments
Source: Trauma Violence Abuse. 2022 Nov 28;24(5):3546–63. doi: 10.1177/15248380221134290 (PMC10594851; doi:10.1177/15248380221134290)
Supplement: sj-docx-1-tva-10.1177_15248380221134290 – Supplemental material for Different Instruments, Same Content? A Systematic Comparison of Child Maltreatment and Harsh Parenting Instruments [file sj-docx-1-tva-10.1177_15248380221134290.docx]

**Appendix A. List of excluded instruments**

Table S1. *List of excluded instruments*

| **Abbr.** | **Instrument full name** | **Source** | **Exclusion reason** |
| --- | --- | --- | --- |
| AAPI-2 | Adult Adolescent Parenting Inventory- Version 2 | Child Maltreatment, Parenting intervention review, Experts | Attitudes not behaviours |
| Angry Outburst | / | Parenting review | No access; not validated instrument |
| APT | Analogue Parenting Task | Child maltreatment review | No clear item structure |
| CAPI | Child Abuse Potential Inventory | Parenting review, Experts | Attitudes and risks not behaviours |
| CNQ | Child Neglect Questionnaire | Child maltreatment review | Neglect measure |
| CNS-MM | Child Neglect Scales – Maternal Monitoring | Child maltreatment review | Neglect measure |
| / | Harsh punishment | Parenting review | No access; not validated instrument |
| / | Harsh discipline | Parenting review | No clear item structure |
| HOME-SF | HOME- Short Form | Expert | Mainly observation |
| / | Ideas about Parenting | Parenting review | No access |
| MCNS | Mother-Child Neglect Scale | Child maltreatment review | Neglect measure |
| MCNS-SF | Mother-Child Neglect Scale- Short Form | Child maltreatment review | Neglect measure |
| NPQ | Nijmengen Parenting Questionnaire | Parenting review | No access |
| PACBM | Parent’s Attributions for Child’s Behavior Measure | Parenting review | Attitudes not behaviours |
| PBI- Child report | Parent Behaviour Inventory | Experts | Not parent self-report |
| P-CAAM | Parent-Child Aggression Acceptability Movie Task | Child maltreatment review | No clear item structure |
| PDR | Parent Daily Report | Parenting review | No clear item structure |
| POQ | Parent Opinion Questionnaire | Child maltreatment review | Measures no harmful parenting behaviours |
| SBS-SV | Shaken Baby Syndrome awareness assessment- Short Version | Child maltreatment review | Baby measure |

**Appendix B. Jaccard Index matrices for harsh parenting and child maltreatment instruments**

Figure 2a. *Jaccard Index Matrix for Harsh Parenting vs Child Maltreatment Instruments - all behaviours*

|  | **APQ** | **BSI** | **CCMS** | **CCNES** | **CTS-ES** | **CTSPC** | **DDI** | **DHS/MICS** | **FM-CA** | **GPBS** | **HDPL** | **HS** | **ICAST-P/-T** | **IPPS** | **JVQ** | **PAFAS** | **PARQ** | **PBC** | **PBI** | **PPI** | **PPS** | **PQ** | **PRCM** | **PS** | **PSDQ** |
| --- | --- | --- | --- | --- | --- | --- | --- | --- | --- | --- | --- | --- | --- | --- | --- | --- | --- | --- | --- | --- | --- | --- | --- | --- | --- |
| **APQ** | - | - | 0.11 | - | 0.00 | 0.25 | - | 0.33 | 0.13 | - | - | - | 0.15 | - | 0.17 | - | - | - | - | - | - | - | - | - | - |
| **BSI** | - | - | 0.14 | - | 0.50 | 0.08 | - | 0.20 | 0.07 | - | - | - | 0.05 | - | 0.25 | - | - | - | - | - | - | - | - | - | - |
| **CCMS** | 0.11 | 0.14 | - | 0.00 | - | - | 0.33 | - | - | 0.25 | 0.45 | 0.25 | - | 0.14 | - | 0.25 | 0.33 | 0.10 | 0.57 | 0.11 | 0.22 | 0.22 | 0.22 | 0.33 | 0.30 |
| **CCNES** | - | - | 0.00 | - | 0.00 | 0.08 | - | 0.20 | 0.00 | - | - | - | 0.05 | - | 0.00 | - | - | - | - | - | - | - | - | - | - |
| **CTS-ES** | 0.00 | 0.50 | - | 0.00 | - | - | 0.00 | - | - | 0.00 | 0.22 | 0.00 | - | 0.00 | - | 0.00 | 0.00 | 0.00 | 0.00 | 0.00 | 0.00 | 0.00 | 0.00 | 0.00 | 0.00 |
| **CTSPC** | 0.25 | 0.08 | - | 0.08 | - | - | 0.21 | - | - | 0.25 | 0.40 | 0.25 | - | 0.08 | - | 0.25 | 0.31 | 0.23 | 0.23 | 0.25 | 0.23 | 0.33 | 0.33 | 0.31 | 0.38 |
| **DDI** | - | - | 0.33 | - | 0.00 | 0.21 | - | 0.25 | 0.25 | - | - | - | 0.19 | - | 0.13 | - | - | - | - | - | - | - | - | - | - |
| **DHS/MICS** | 0.33 | 0.20 | - | 0.20 | - | - | 0.25 | - | - | 0.33 | 0.40 | 0.33 | - | 0.20 | - | 0.60 | 0.43 | 0.29 | 0.29 | 0.33 | 0.29 | 0.29 | 0.29 | 0.43 | 0.22 |
| **FM-CA** | 0.13 | 0.07 | - | 0.00 | - | - | 0.25 | - | - | 0.20 | 0.33 | 0.13 | - | 0.07 | - | 0.13 | 0.18 | 0.12 | 0.27 | 0.13 | 0.12 | 0.19 | 0.19 | 0.18 | 0.31 |
| **GPBS** | - | - | 0.25 | - | 0.00 | 0.25 | - | 0.33 | 0.20 | - | - | - | 0.15 | - | 0.17 | - | - | - | - | - | - | - | - | - | - |
| **HDPL** | - | - | 0.45 | - | 0.22 | 0.40 | - | 0.40 | 0.33 | - | - | - | 0.45 | - | 0.44 | - | - | - | - | - | - | - | - | - | - |
| **HS** | - | - | 0.25 | - | 0.00 | 0.25 | - | 0.33 | 0.13 | - | - | - | 0.15 | - | 0.17 | - | - | - | - | - | - | - | - | - | - |
| **ICAST-P/-T** | 0.15 | 0.05 | - | 0.05 | - | - | 0.19 | - | - | 0.15 | 0.45 | 0.15 | - | 0.05 | - | 0.15 | 0.25 | 0.20 | 0.14 | 0.15 | 0.20 | 0.20 | 0.20 | 0.19 | 0.18 |
| **IPPS** | - | - | 0.14 | - | 0.00 | 0.08 | - | 0.20 | 0.07 | - | - | - | 0.05 | - | 0.25 | - | - | - | - | - | - | - | - | - | - |
| **JVQ** | 0.17 | 0.25 | - | 0.00 | - | - | 0.13 | - | - | 0.17 | 0.44 | 0.17 | - | 0.25 | - | 0.40 | 0.29 | 0.14 | 0.33 | 0.17 | 0.14 | 0.14 | 0.14 | 0.29 | 0.11 |
| **PAFAS** | - | - | 0.25 | - | 0.00 | 0.25 | - | 0.60 | 0.13 | - | - | - | 0.15 | - | 0.40 | - | - | - | - | - | - | - | - | - | - |
| **PARQ** | - | - | 0.33 | - | 0.00 | 0.31 | - | 0.43 | 0.18 | - | - | - | 0.25 | - | 0.29 | - | - | - | - | - | - | - | - | - | - |
| **PBC** | - | - | 0.10 | - | 0.00 | 0.23 | - | 0.29 | 0.12 | - | - | - | 0.20 | - | 0.14 | - | - | - | - | - | - | - | - | - | - |
| **PBI** | - | - | 0.57 | - | 0.00 | 0.23 | - | 0.29 | 0.27 | - | - | - | 0.14 | - | 0.33 | - | - | - | - | - | - | - | - | - | - |
| **PPI** | - | - | 0.11 | - | 0.00 | 0.25 | - | 0.33 | 0.13 | - | - | - | 0.15 | - | 0.17 | - | - | - | - | - | - | - | - | - | - |
| **PPS** | - | - | 0.22 | - | 0.00 | 0.23 | - | 0.29 | 0.12 | - | - | - | 0.20 | - | 0.14 | - | - | - | - | - | - | - | - | - | - |
| **PQ** | - | - | 0.22 | - | 0.00 | 0.33 | - | 0.29 | 0.19 | - | - | - | 0.20 | - | 0.14 | - | - | - | - | - | - | - | - | - | - |
| **PRCM** | - | - | 0.22 | - | 0.00 | 0.33 | - | 0.29 | 0.19 | - | - | - | 0.20 | - | 0.14 | - | - | - | - | - | - | - | - | - | - |
| **PS** | - | - | 0.33 | - | 0.00 | 0.31 | - | 0.43 | 0.18 | - | - | - | 0.19 | - | 0.29 | - | - | - | - | - | - | - | - | - | - |
| **PSDQ** | - | - | 0.30 | - | 0.00 | 0.38 | - | 0.22 | 0.31 | - | - | - | 0.18 | - | 0.11 | - | - | - | - | - | - | - | - | - | - |
| **Overall** | 0.16 | 0.18 | 0.24 | 0.05 | 0.04 | 0.25 | 0.19 | 0.32 | 0.16 | 0.19 | 0.39 | 0.18 | 0.17 | 0.11 | 0.21 | 0.25 | 0.25 | 0.15 | 0.26 | 0.16 | 0.17 | 0.20 | 0.20 | 0.25 | 0.22 |

* Colour-coding: red = very weak Jaccard Index (0.00 - 0.19), orange = weak Jaccard Index (0.20 - 0.39), yellow = moderate Jaccard Index (0.40-0.59), light green = strong Jaccard Index (0.60 - 0.79), dark green = very strong Jaccard Index (0.80 - 1.0).

Figure 2b*. Jaccard Index Matrix for Harsh Parenting vs Child Maltreatment Instruments - physical behaviours*

|  | **APQ** | **BSI** | **CCMS** | **CTS-ES** | **CTSPC** | **DDI** | **DHS/MICS** | **FM-CA** | **GPBS** | **HDPL** | **HS** | **ICAST-P/T** | **IPPS** | **JVQ** | **PAFAS** | **PARQ** | **PBC** | **PBI** | **PPI** | **PPS** | **PQ** | **PRCM** | **PS** | **PSDQ** |
| --- | --- | --- | --- | --- | --- | --- | --- | --- | --- | --- | --- | --- | --- | --- | --- | --- | --- | --- | --- | --- | --- | --- | --- | --- |
| **APQ** | - | - | 0.17 | 0.00 | 0.25 | - | 0.25 | 0.15 | - | - | - | 0.13 | - | 0.25 | - | - | - | - | - | - | - | - | - | - |
| **BSI** | - | - | 0.20 | 0.50 | 0.13 | - | 0.33 | 0.08 | - | - | - | 0.07 | - | 0.33 | - | - | - | - | - | - | - | - | - | - |
| **CCMS** | 0.17 | 0.20 | - | - | - | 0.43 | - | - | 0.33 | 0.38 | 0.20 | - | 0.20 | - | 0.20 | 0.20 | 0.17 | 0.40 | 0.17 | 0.20 | 0.17 | 0.17 | 0.40 | 0.29 |
| **CTS-ES** | 0.00 | 0.50 | - | - | - | 0.00 | - | - | 0.00 | 0.33 | 0.00 | - | 0.00 | - | 0.00 | 0.00 | 0.00 | 0.00 | 0.00 | 0.00 | 0.00 | 0.00 | 0.00 | 0.00 |
| **CTSPC** | 0.25 | 0.13 | - | - | - | 0.30 | - | - | 0.38 | 0.27 | 0.13 | - | 0.13 | - | 0.13 | 0.13 | 0.25 | 0.11 | 0.25 | 0.13 | 0.25 | 0.25 | 0.11 | 0.33 |
| **DDI** | - | - | 0.43 | 0.00 | 0.30 | - | 0.33 | 0.29 | - | - | - | 0.25 | - | 0.14 | - | - | - | - | - | - | - | - | - | - |
| **DHS/MICS** | 0.25 | 0.33 | - | - | - | 0.33 | - | - | 0.50 | 0.29 | 0.33 | - | 0.33 | - | 0.33 | 0.33 | 0.25 | 0.25 | 0.25 | 0.33 | 0.25 | 0.25 | 0.25 | 0.17 |
| **FM-CA** | 0.15 | 0.08 | - | - | - | 0.29 | - | - | 0.23 | 0.27 | 0.08 | - | 0.08 | - | 0.08 | 0.08 | 0.15 | 0.15 | 0.15 | 0.08 | 0.15 | 0.15 | 0.15 | 0.31 |
| **GPBS** | - | - | 0.33 | 0.00 | 0.38 | - | 0.50 | 0.23 | 0.00 | - | - | 0.20 | - | 0.20 | - | - | - | - | - | - | - | - | - | - |
| **HDPL** | - | - | 0.38 | 0.33 | 0.27 | - | 0.29 | 0.27 | - | - | - | 0.40 | - | 0.50 | - | - | - | - | - | - | - | - | - | - |
| **HS** | - | - | 0.20 | 0.00 | 0.13 | - | 0.33 | 0.08 | - | - | - | 0.07 | - | 0.33 | - | - | - | - | - | - | - | - | - | - |
| **ICAST-P/T** | 0.13 | 0.07 | - | - | - | 0.25 | - | - | 0.20 | 0.40 | 0.07 | - | 0.07 | - | 0.07 | 0.07 | 0.13 | 0.06 | 0.13 | 0.07 | 0.13 | 0.13 | 0.06 | 0.12 |
| **IPPS** | - | - | 0.20 | 0.00 | 0.13 | - | 0.33 | 0.08 | - | - | - | 0.07 | - | 0.33 | - | - | - | - | - | - | - | - | - | - |
| **JVQ** | 0.25 | 0.33 | - | - | - | 0.14 | - | - | 0.20 | 0.50 | 0.33 | - | 0.33 | - | 0.33 | 0.33 | 0.25 | 0.25 | 0.25 | 0.33 | 0.25 | 0.25 | 0.25 | 0.17 |
| **PAFAS** | - | - | 0.20 | 0.00 | 0.13 | - | 0.33 | 0.08 | - | - | - | 0.07 | - | 0.33 | - | - | - | - | - | - | - | - | - | - |
| **PARQ** | - | - | 0.20 | 0.00 | 0.13 | - | 0.33 | 0.08 | - | - | - | 0.07 | - | 0.33 | - | - | - | - | - | - | - | - | - | - |
| **PBC** | - | - | 0.17 | 0.00 | 0.25 | - | 0.25 | 0.15 | - | - | - | 0.13 | - | 0.25 | - | - | - | - | - | - | - | - | - | - |
| **PBI** | - | - | 0.40 | 0.00 | 0.11 | - | 0.25 | 0.15 | - | - | - | 0.06 | - | 0.25 | - | - | - | - | - | - | - | - | - | - |
| **PPI** | - | - | 0.17 | 0.00 | 0.25 | - | 0.25 | 0.15 | - | - | - | 0.13 | - | 0.25 | - | - | - | - | - | - | - | - | - | - |
| **PPS** | - | - | 0.20 | 0.00 | 0.13 | - | 0.33 | 0.08 | - | - | - | 0.07 | - | 0.33 | - | - | - | - | - | - | - | - | - | - |
| **PQ** | - | - | 0.17 | 0.00 | 0.25 | - | 0.25 | 0.15 | - | - | - | 0.13 | - | 0.25 | - | - | - | - | - | - | - | - | - | - |
| **PRCM** | - | - | 0.17 | 0.00 | 0.25 | - | 0.25 | 0.15 | - | - | - | 0.13 | - | 0.25 | - | - | - | - | - | - | - | - | - | - |
| **PS** | - | - | 0.40 | 0.00 | 0.11 | - | 0.25 | 0.15 | - | - | - | 0.06 | - | 0.25 | - | - | - | - | - | - | - | - | - | - |
| **PSDQ** | - | - | 0.29 | 0.00 | 0.33 | - | 0.17 | 0.31 | - | - | - | 0.12 | - | 0.17 | - | - | - | - | - | - | - | - | - | - |
| **Overall** | 0.17 | 0.23 | 0.25 | 0.05 | 0.21 | 0.25 | 0.30 | 0.15 | 0.23 | 0.35 | 0.16 | 0.13 | 0.16 | 0.28 | 0.16 | 0.16 | 0.17 | 0.18 | 0.17 | 0.16 | 0.17 | 0.17 | 0.18 | 0.20 |

* Colour-coding: red = very weak Jaccard Index (0.00 - 0.19), orange = weak Jaccard Index (0.20 - 0.39), yellow = moderate Jaccard Index (0.40-0.59), light green = strong Jaccard Index (0.60 - 0.79), dark green = very strong Jaccard Index (0.80 - 1.0).

Figure 2c. *Jaccard Index Matrix for Harsh Parenting vs Child Maltreatment Instruments - emotional behaviours*

|  | **APQ** | **CCMS** | **CCNES** | **CTSPC** | **DDI** | **DHS/MICS** | **FM-CA** | **HDPL** | **HS** | **ICAST-P/T** | **JVQ** | **PAFAS** | **PARQ** | **PBC** | **PBI** | **PPI** | **PPS** | **PQ** | **PRCM** | **PS** | **PSDQ** |
| --- | --- | --- | --- | --- | --- | --- | --- | --- | --- | --- | --- | --- | --- | --- | --- | --- | --- | --- | --- | --- | --- |
| **APQ** | - | 0.00 | - | 0.25 | - | 0.50 | 0.00 | - | - | 0.20 | 0.00 | - | - | - | - | - | - | - | - | - | - |
| **CCMS** | 0.00 | - | 0.00 | - | 0.25 | - | - | 0.67 | 0.33 | - | - | 0.33 | 0.50 | 0.00 | 1.00 | 0.00 | 0.25 | 0.33 | 0.33 | 0.25 | 0.33 |
| **CCNES** | - | 0.00 | - | 0.25 | - | 0.50 | 0.00 | - | - | 0.20 | 0.00 | - | - | - | - | - | - | - | - | - | - |
| **CTSPC** | 0.25 | - | 0.25 | - | 0.40 | - | - | 0.75 | 0.50 | - | - | 0.50 | 0.60 | 0.20 | 0.50 | 0.25 | 0.40 | 0.50 | 0.50 | 0.75 | 0.50 |
| **DDI** | - | 0.25 | - | 0.40 | - | 0.67 | 0.25 | - | - | 0.60 | 0.33 | - | - | - | - | - | - | - | - | - | - |
| **DHS/MICS** | 0.50 | - | 0.50 | - | 0.67 | - | - | 0.67 | 0.33 | - | - | 1.00 | 0.50 | 0.33 | 0.33 | 0.50 | 0.25 | 0.33 | 0.33 | 0.67 | 0.33 |
| **FM-CA** | 0.00 | - | 0.00 | - | 0.25 | - | - | 0.67 | 0.33 | - | - | 0.33 | 0.50 | 0.00 | 1.00 | 0.00 | 0.25 | 0.33 | 0.33 | 0.25 | 0.33 |
| **HDPL** | - | 0.67 | - | 0.75 | - | 0.67 | 0.67 | - | - | 0.60 | 0.33 | - | - | - | - | - | - | - | - | - | - |
| **HS** | - | 0.33 | - | 0.50 | - | 0.33 | 0.33 | - | - | 0.40 | 0.00 | - | - | - | - | - | - | - | - | - | - |
| **ICAST-P/T** | 0.20 | - | 0.20 | - | 0.60 | - | - | 0.60 | 0.40 | - | - | 0.40 | 0.80 | 0.40 | 0.40 | 0.20 | 0.60 | 0.40 | 0.40 | 0.60 | 0.40 |
| **JVQ** | 0.00 | - | 0.00 | - | 0.33 | - | - | 0.33 | 0.00 | - | - | 0.50 | 0.25 | 0.00 | 0.50 | 0.00 | 0.00 | 0.00 | 0.00 | 0.33 | 0.00 |
| **PAFAS** | - | 0.33 | - | 0.50 | - | 1.00 | 0.33 | - | - | 0.40 | 0.50 | - | - | - | - | - | - | - | - | - | - |
| **PARQ** | - | 0.50 | - | 0.60 | - | 0.50 | 0.50 | - | - | 0.80 | 0.25 | - | - | - | - | - | - | - | - | - | - |
| **PBC** | - | 0.00 | - | 0.20 | - | 0.33 | 0.00 | - | - | 0.40 | 0.00 | - | - | - | - | - | - | - | - | - | - |
| **PBI** | - | 1.00 | - | 0.50 | - | 0.33 | 1.00 | - | - | 0.40 | 0.50 | - | - | - | - | - | - | - | - | - | - |
| **PPI** | - | 0.00 | - | 0.25 | - | 0.50 | 0.00 | - | - | 0.20 | 0.00 | - | - | - | - | - | - | - | - | - | - |
| **PPS** | - | 0.25 | - | 0.40 | - | 0.25 | 0.25 | - | - | 0.60 | 0.00 | - | - | - | - | - | - | - | - | - | - |
| **PQ** | - | 0.33 | - | 0.50 | - | 0.33 | 0.33 | - | - | 0.40 | 0.00 | - | - | - | - | - | - | - | - | - | - |
| **PRCM** | - | 0.33 | - | 0.50 | - | 0.33 | 0.33 | - | - | 0.40 | 0.00 | - | - | - | - | - | - | - | - | - | - |
| **PS** | - | 0.25 | - | 0.75 | - | 0.67 | 0.25 | - | - | 0.60 | 0.33 | - | - | - | - | - | - | - | - | - | - |
| **PSDQ** | - | 0.33 | - | 0.50 | - | 0.33 | 0.33 | - | - | 0.40 | 0.00 | - | - | - | - | - | - | - | - | - | - |
| **Overall** | **0.16** | **0.31** | **0.16** | **0.46** | **0.42** | **0.48** | **0.31** | **0.61** | **0.32** | **0.44** | **0.15** | **0.51** | **0.53** | **0.16** | **0.62** | **0.16** | **0.29** | **0.32** | **0.32** | **0.48** | **0.32** |

* Colour-coding: red = very weak Jaccard Index (0.00 - 0.19), orange = weak Jaccard Index (0.20 - 0.39), yellow = moderate Jaccard Index (0.40-0.59), light green = strong Jaccard Index (0.60 - 0.79), dark green = very strong Jaccard Index (0.80 - 1.0).

Figure 3a. *Jaccard Index Matrix for Child Maltreatment Instruments - all behaviours*

|  | **CCMS** | **CTS-ES** | **CTSPC** | **DHS/MICS** | **FM-CA** | **ICAST-P/-T** | **JVQ** |
| --- | --- | --- | --- | --- | --- | --- | --- |
| **CCMS** | / | 0.29 | 0.36 | 0.50 | 0.47 | 0.29 | 0.57 |
| **CTS-ES** | 0.29 | / | 0.08 | 0.17 | 0.13 | 0.10 | 0.50 |
| **CTSPC** | 0.36 | 0.08 | / | 0.42 | 0.59 | 0.52 | 0.23 |
| **DHS/MICS** | 0.50 | 0.17 | 0.42 | / | 0.25 | 0.25 | 0.50 |
| **FM-CA** | 0.47 | 0.13 | 0.59 | 0.25 | / | 0.59 | 0.27 |
| **ICAST-P/-T** | 0.29 | 0.10 | 0.52 | 0.25 | 0.59 | / | 0.20 |
| **JVQ** | 0.57 | 0.50 | 0.23 | 0.50 | 0.27 | 0.20 | / |
| **Overall** | **0.41** | **0.21** | **0.37** | **0.35** | **0.38** | **0.32** | **0.38** |

* Colour-coding: red = very weak Jaccard Index (0.00 - 0.19), orange = weak Jaccard Index (0.20 - 0.39), yellow = moderate Jaccard Index (0.40-0.59), light green = strong Jaccard Index (0.60 - 0.79), dark green = very strong Jaccard Index (0.80 - 1.0).

Figure 3b. *Jaccard Index Matrix for Child Maltreatment Instruments - physical behaviours*

|  | **CCMS** | **CTS-ES** | **CTSPC** | **DHS/MICS** | **FM-CA** | **ICAST-P/T** | **JVQ** |
| --- | --- | --- | --- | --- | --- | --- | --- |
| **CCMS** | / | 0.40 | 0.30 | 0.60 | 0.38 | 0.25 | 0.60 |
| **CTS-ES** | 0.40 | / | 0.11 | 0.25 | 0.15 | 0.13 | 0.67 |
| **CTSPC** | 0.30 | 0.11 | / | 0.38 | 0.62 | 0.44 | 0.22 |
| **DHS/MICS** | 0.60 | 0.25 | 0.38 | / | 0.23 | 0.20 | 0.50 |
| **FM-CA** | 0.38 | 0.15 | 0.62 | 0.23 | / | 0.65 | 0.23 |
| **ICAST-P/T** | 0.25 | 0.13 | 0.44 | 0.20 | 0.65 | / | 0.20 |
| **JVQ** | 0.60 | 0.67 | 0.22 | 0.50 | 0.23 | 0.20 | / |
| **Overall** | **0.42** | **0.29** | **0.34** | **0.36** | **0.38** | **0.31** | **0.40** |

* Colour-coding: red = very weak Jaccard Index (0.00 - 0.19), orange = weak Jaccard Index (0.20 - 0.39), yellow = moderate Jaccard Index (0.40-0.59), light green = strong Jaccard Index (0.60 - 0.79), dark green = very strong Jaccard Index (0.80 - 1.0).

Figure 3c. *Jaccard Index Matrix for Child Maltreatment Instruments - emotional behaviours*

|  | **CCMS** | **CTSPC** | **DHS/MICS** | **FM-CA** | **ICAST-P/T** | **JVQ** |
| --- | --- | --- | --- | --- | --- | --- |
| **CCMS** | / | 0.50 | 0.33 | 1.00 | 0.40 | 0.50 |
| **CTSPC** | 0.50 | / | 0.50 | 0.50 | 0.80 | 0.25 |
| **DHS/MICS** | 0.33 | 0.50 | / | 0.33 | 0.40 | 0.50 |
| **FM-CA** | 1.00 | 0.50 | 0.33 | / | 0.40 | 0.50 |
| **ICAST-P/T** | 0.40 | 0.80 | 0.40 | 0.40 | / | 0.20 |
| **JVQ** | 0.50 | 0.25 | 0.50 | 0.50 | 0.20 | / |
| **Overall** | **0.55** | **0.51** | **0.41** | **0.55** | **0.44** | **0.39** |

* Colour-coding: red = very weak Jaccard Index (0.00 - 0.19), orange = weak Jaccard Index (0.20 - 0.39), yellow = moderate Jaccard Index (0.40-0.59), light green = strong Jaccard Index (0.60 - 0.79), dark green = very strong Jaccard Index (0.80 - 1.0).

Figure 4a*. Jaccard Index Matrix for Harsh Parenting Instruments - all behaviours*

|  | **APQ** | **BSI** | **CCNES** | **DDI** | **GPBS** | **HDPL** | **HS** | **IPPS** | **PAFAS** | **PARQ** | **PBC** | **PBI** | **PPI** | **PPS** | **PQ** | **PRCM** | **PS** | **PSDQ** |
| --- | --- | --- | --- | --- | --- | --- | --- | --- | --- | --- | --- | --- | --- | --- | --- | --- | --- | --- |
| **APQ** | / | 0.00 | 0.33 | 0.33 | 0.50 | 0.20 | 0.50 | 0.33 | 0.50 | 0.33 | 0.75 | 0.17 | 1.00 | 0.40 | 0.75 | 0.75 | 0.33 | 0.50 |
| **BSI** | 0.00 | / | 0.00 | 0.00 | 0.00 | 0.11 | 0.00 | 0.00 | 0.00 | 0.00 | 0.00 | 0.00 | 0.00 | 0.00 | 0.00 | 0.00 | 0.00 | 0.00 |
| **CCNES** | 0.33 | 0.00 | / | 0.00 | 0.00 | 0.11 | 0.33 | 0.00 | 0.33 | 0.20 | 0.25 | 0.00 | 0.33 | 0.25 | 0.25 | 0.25 | 0.20 | 0.17 |
| **DDI** | 0.33 | 0.00 | 0.00 | / | 0.60 | 0.08 | 0.14 | 0.20 | 0.14 | 0.11 | 0.29 | 0.29 | 0.33 | 0.13 | 0.29 | 0.29 | 0.25 | 0.38 |
| **GPBS** | 0.50 | 0.00 | 0.00 | 0.60 | / | 0.09 | 0.20 | 0.33 | 0.20 | 0.14 | 0.40 | 0.17 | 0.50 | 0.17 | 0.40 | 0.40 | 0.14 | 0.29 |
| **HDPL** | 0.20 | 0.11 | 0.11 | 0.08 | 0.09 | / | 0.33 | 0.11 | 0.33 | 0.40 | 0.18 | 0.30 | 0.20 | 0.30 | 0.30 | 0.30 | 0.27 | 0.25 |
| **HS** | 0.50 | 0.00 | 0.33 | 0.14 | 0.20 | 0.33 | / | 0.33 | 0.50 | 0.60 | 0.40 | 0.40 | 0.50 | 0.75 | 0.75 | 0.75 | 0.33 | 0.50 |
| **IPPS** | 0.33 | 0.00 | 0.00 | 0.20 | 0.33 | 0.11 | 0.33 | / | 0.33 | 0.20 | 0.25 | 0.25 | 0.33 | 0.25 | 0.25 | 0.25 | 0.20 | 0.17 |
| **PAFAS** | 0.50 | 0.00 | 0.33 | 0.14 | 0.20 | 0.33 | 0.50 | 0.33 | / | 0.60 | 0.40 | 0.40 | 0.50 | 0.40 | 0.40 | 0.40 | 0.60 | 0.29 |
| **PARQ** | 0.33 | 0.00 | 0.20 | 0.11 | 0.14 | 0.40 | 0.60 | 0.20 | 0.60 | / | 0.50 | 0.50 | 0.33 | 0.80 | 0.50 | 0.50 | 0.43 | 0.38 |
| **PBC** | 0.75 | 0.00 | 0.25 | 0.29 | 0.40 | 0.18 | 0.40 | 0.25 | 0.40 | 0.50 | / | 0.14 | 0.75 | 0.60 | 0.60 | 0.60 | 0.29 | 0.43 |
| **PBI** | 0.17 | 0.00 | 0.00 | 0.29 | 0.17 | 0.30 | 0.40 | 0.25 | 0.40 | 0.50 | 0.14 | / | 0.17 | 0.33 | 0.33 | 0.33 | 0.50 | 0.43 |
| **PPI** | 1.00 | 0.00 | 0.33 | 0.33 | 0.50 | 0.20 | 0.50 | 0.33 | 0.50 | 0.33 | 0.75 | 0.17 | / | 0.40 | 0.75 | 0.75 | 0.33 | 0.50 |
| **PPS** | 0.40 | 0.00 | 0.25 | 0.13 | 0.17 | 0.30 | 0.75 | 0.25 | 0.40 | 0.80 | 0.60 | 0.33 | 0.40 | / | 0.60 | 0.60 | 0.29 | 0.43 |
| **PQ** | 0.75 | 0.00 | 0.25 | 0.29 | 0.40 | 0.30 | 0.75 | 0.25 | 0.40 | 0.50 | 0.60 | 0.33 | 0.75 | 0.60 | / | 1.00 | 0.29 | 0.67 |
| **PRCM** | 0.75 | 0.00 | 0.25 | 0.29 | 0.40 | 0.30 | 0.75 | 0.25 | 0.40 | 0.50 | 0.60 | 0.33 | 0.75 | 0.60 | 1.00 | / | 0.29 | 0.67 |
| **PS** | 0.33 | 0.00 | 0.20 | 0.25 | 0.14 | 0.27 | 0.33 | 0.20 | 0.60 | 0.43 | 0.29 | 0.50 | 0.33 | 0.29 | 0.29 | 0.29 | / | 0.38 |
| **PSDQ** | 0.50 | 0.00 | 0.17 | 0.38 | 0.29 | 0.25 | 0.50 | 0.17 | 0.29 | 0.38 | 0.43 | 0.43 | 0.50 | 0.43 | 0.67 | 0.67 | 0.38 | / |
| **Overall** | **0.45** | **0.01** | **0.18** | **0.23** | **0.27** | **0.23** | **0.43** | **0.22** | **0.37** | **0.38** | **0.40** | **0.28** | **0.45** | **0.39** | **0.48** | **0.48** | **0.30** | **0.38** |

* Colour-coding: red = very weak Jaccard Index (0.00 - 0.19), orange = weak Jaccard Index (0.20 - 0.39), yellow = moderate Jaccard Index (0.40-0.59), light green = strong Jaccard Index (0.60 - 0.79), dark green = very strong Jaccard Index (0.80 - 1.0).

Figure 4b. *Jaccard Index Matrix for Harsh Parenting Instruments - physical behaviours*

|  | **APQ** | **BSI** | **DDI** | **GPBS** | **HDPL** | **HS** | **IPPS** | **PAFAS** | **PARQ** | **PBC** | **PBI** | **PPI** | **PPS** | **PQ** | **PRCM** | **PS** | **PSDQ** |
| --- | --- | --- | --- | --- | --- | --- | --- | --- | --- | --- | --- | --- | --- | --- | --- | --- | --- |
| **APQ** | / | 0.00 | 0.40 | 0.67 | 0.14 | 0.50 | 0.50 | 0.50 | 0.50 | 1.00 | 0.33 | 1.00 | 0.50 | 1.00 | 1.00 | 0.33 | 0.50 |
| **BSI** | 0.00 | / | 0.00 | 0.00 | 0.17 | 0.00 | 0.00 | 0.00 | 0.00 | 0.00 | 0.00 | 0.00 | 0.00 | 0.00 | 0.00 | 0.00 | 0.00 |
| **DDI** | 0.40 | 0.00 | / | 0.60 | 0.10 | 0.20 | 0.20 | 0.20 | 0.20 | 0.40 | 0.40 | 0.40 | 0.20 | 0.40 | 0.40 | 0.40 | 0.40 |
| **GPBS** | 0.67 | 0.00 | 0.60 | / | 0.13 | 0.33 | 0.33 | 0.33 | 0.33 | 0.67 | 0.25 | 0.67 | 0.33 | 0.67 | 0.67 | 0.25 | 0.40 |
| **HDPL** | 0.14 | 0.17 | 0.10 | 0.13 | / | 0.17 | 0.17 | 0.17 | 0.17 | 0.14 | 0.14 | 0.14 | 0.17 | 0.14 | 0.14 | 0.14 | 0.11 |
| **HS** | 0.50 | 0.00 | 0.20 | 0.33 | 0.17 | / | 1.00 | 1.00 | 1.00 | 0.50 | 0.50 | 0.50 | 1.00 | 0.50 | 0.50 | 0.50 | 0.25 |
| **IPPS** | 0.50 | 0.00 | 0.20 | 0.33 | 0.17 | 1.00 | / | 1.00 | 1.00 | 0.50 | 0.50 | 0.50 | 1.00 | 0.50 | 0.50 | 0.50 | 0.25 |
| **PAFAS** | 0.50 | 0.00 | 0.20 | 0.33 | 0.17 | 1.00 | 1.00 | / | 1.00 | 0.50 | 0.50 | 0.50 | 1.00 | 0.50 | 0.50 | 0.50 | 0.25 |
| **PARQ** | 0.50 | 0.00 | 0.20 | 0.33 | 0.17 | 1.00 | 1.00 | 1.00 | / | 0.50 | 0.50 | 0.50 | 1.00 | 0.50 | 0.50 | 0.50 | 0.25 |
| **PBC** | 1.00 | 0.00 | 0.40 | 0.67 | 0.14 | 0.50 | 0.50 | 0.50 | 0.50 | / | 0.33 | 1.00 | 0.50 | 1.00 | 1.00 | 0.33 | 0.50 |
| **PBI** | 0.33 | 0.00 | 0.40 | 0.25 | 0.14 | 0.50 | 0.50 | 0.50 | 0.50 | 0.33 | / | 0.33 | 0.50 | 0.33 | 0.33 | 1.00 | 0.50 |
| **PPI** | 1.00 | 0.00 | 0.40 | 0.67 | 0.14 | 0.50 | 0.50 | 0.50 | 0.50 | 1.00 | 0.33 | / | 0.50 | 1.00 | 1.00 | 0.33 | 0.50 |
| **PPS** | 0.50 | 0.00 | 0.20 | 0.33 | 0.17 | 1.00 | 1.00 | 1.00 | 1.00 | 0.50 | 0.50 | 0.50 | / | 0.50 | 0.50 | 0.50 | 0.25 |
| **PQ** | 1.00 | 0.00 | 0.40 | 0.67 | 0.14 | 0.50 | 0.50 | 0.50 | 0.50 | 1.00 | 0.33 | 1.00 | 0.50 | / | 1.00 | 0.33 | 0.50 |
| **PRCM** | 1.00 | 0.00 | 0.40 | 0.67 | 0.14 | 0.50 | 0.50 | 0.50 | 0.50 | 1.00 | 0.33 | 1.00 | 0.50 | 1.00 | / | 0.33 | 0.33 |
| **PS** | 0.33 | 0.00 | 0.40 | 0.25 | 0.14 | 0.50 | 0.50 | 0.50 | 0.50 | 0.33 | 1.00 | 0.33 | 0.50 | 0.33 | 0.33 | / | 0.50 |
| **PSDQ** | 0.50 | 0.00 | 0.50 | 0.40 | 0.11 | 0.25 | 0.33 | 0.25 | 0.25 | 0.50 | 0.50 | 0.50 | 0.25 | 0.50 | 0.50 | 0.50 | / |
| **Overall** | 0.52 | 0.01 | 0.29 | 0.39 | 0.14 | 0.56 | 0.50 | 0.50 | 0.50 | 0.52 | 0.38 | 0.52 | 0.50 | 0.52 | 0.52 | 0.38 | 0.34 |

* Colour-coding: red = very weak Jaccard Index (0.00 - 0.19), orange = weak Jaccard Index (0.20 - 0.39), yellow = moderate Jaccard Index (0.40-0.59), light green = strong Jaccard Index (0.60 - 0.79), dark green = very strong Jaccard Index (0.80 - 1.0).

Figure 4c. *Jaccard Index Matrix for Harsh Parenting Instruments - emotional behaviours*

|  | **APQ** | **CCNES** | **DDI** | **HDPL** | **HS** | **PAFAS** | **PARQ** | **PBC** | **PBI** | **PPI** | **PPS** | **PQ** | **PRCM** | **PS** | **PSDQ** |
| --- | --- | --- | --- | --- | --- | --- | --- | --- | --- | --- | --- | --- | --- | --- | --- |
| **APQ** | / | 1.00 | 0.33 | 0.33 | 0.50 | 0.50 | 0.25 | 0.50 | 0.00 | 1.00 | 0.33 | 0.50 | 0.50 | 0.33 | 0.50 |
| **CCNES** | 1.00 | / | 0.33 | 0.33 | 0.50 | 0.50 | 0.25 | 0.50 | 0.00 | 1.00 | 0.33 | 0.50 | 0.50 | 0.33 | 0.50 |
| **DDI** | 0.33 | 0.33 | / | 0.50 | 0.25 | 0.67 | 0.75 | 0.67 | 0.25 | 0.33 | 0.50 | 0.25 | 0.25 | 0.50 | 0.25 |
| **HDPL** | 0.33 | 0.33 | 0.50 | / | 0.67 | 0.67 | 0.75 | 0.25 | 0.67 | 0.33 | 0.50 | 0.67 | 0.67 | 0.50 | 0.67 |
| **HS** | 0.50 | 0.50 | 0.25 | 0.67 | / | 0.33 | 0.50 | 0.33 | 0.33 | 0.50 | 0.67 | 1.00 | 1.00 | 0.25 | 1.00 |
| **PAFAS** | 0.50 | 0.50 | 0.67 | 0.67 | 0.33 | / | 0.50 | 0.33 | 0.33 | 0.50 | 0.25 | 0.33 | 0.33 | 0.67 | 0.33 |
| **PARQ** | 0.25 | 0.25 | 0.75 | 0.75 | 0.50 | 0.50 | / | 0.50 | 0.50 | 0.25 | 0.75 | 0.50 | 0.50 | 0.40 | 0.50 |
| **PBC** | 0.50 | 0.50 | 0.67 | 0.25 | 0.33 | 0.33 | 0.50 | / | 0.00 | 0.50 | 0.67 | 0.33 | 0.33 | 0.25 | 0.33 |
| **PBI** | 0.00 | 0.00 | 0.25 | 0.67 | 0.33 | 0.33 | 0.50 | 0.00 | / | 0.00 | 0.25 | 0.33 | 0.33 | 0.25 | 0.33 |
| **PPI** | 1.00 | 1.00 | 0.33 | 0.33 | 0.50 | 0.50 | 0.25 | 0.50 | 0.00 | / | 0.33 | 0.50 | 0.50 | 0.33 | 0.50 |
| **PPS** | 0.33 | 0.33 | 0.50 | 0.50 | 0.67 | 0.25 | 0.75 | 0.67 | 0.25 | 0.33 | / | 0.67 | 0.67 | 0.20 | 0.67 |
| **PQ** | 0.50 | 0.50 | 0.25 | 0.67 | 1.00 | 0.33 | 0.50 | 0.33 | 0.33 | 0.50 | 0.67 | / | 1.00 | 0.25 | 1.00 |
| **PRCM** | 0.50 | 0.50 | 0.25 | 0.67 | 1.00 | 0.33 | 0.50 | 0.33 | 0.33 | 0.50 | 0.67 | 1.00 | / | 0.25 | 1.00 |
| **PS** | 0.33 | 0.33 | 0.50 | 0.50 | 0.25 | 0.67 | 0.40 | 0.25 | 0.25 | 0.33 | 0.20 | 0.25 | 0.25 | / | 0.25 |
| **PSDQ** | 0.50 | 0.50 | 0.25 | 0.67 | 1.00 | 0.33 | 0.50 | 0.33 | 0.33 | 0.50 | 0.67 | 1.00 | 1.00 | 0.25 | / |
| **Overall** | 0.47 | 0.47 | 0.42 | 0.54 | 0.56 | 0.45 | 0.49 | 0.39 | 0.26 | 0.47 | 0.48 | 0.56 | 0.56 | 0.34 | 0.56 |

* Colour-coding: red = very weak Jaccard Index (0.00 - 0.19), orange = weak Jaccard Index (0.20 - 0.39), yellow = moderate Jaccard Index (0.40-0.59), light green = strong Jaccard Index (0.60 - 0.79), dark green = very strong Jaccard Index (0.80 - 1.0).
